# Safety and efficacy of sorafenib therapy in patients with hepatocellular carcinoma: final outcome from the Chinese patient subset of the GIDEON study

**DOI:** 10.18632/oncotarget.6781

**Published:** 2015-12-28

**Authors:** Sheng-Long Ye, Xiaoping Chen, Jiamei Yang, Ping Bie, Shuijun Zhang, Fengyong Liu, Luming Liu, Jie Zhou, Kefeng Dou, Chunyi Hao, Guoliang Shao, Qiang Xia, Yajin Chen, Jijin Yang, Xiaxing Deng, Yunpeng Liu, Yunfei Yuan, Zhiren Fu, Keiko Nakajima, Christina S.M. Yip, Zhengguang Lu

**Affiliations:** ^1^ Liver Cancer Institute, Zhongshan Hospital, Fudan University, Shanghai, China; ^2^ Department of Surgery, Tongji Medical College, Huazhong University of Science and Technology, Wuhan, China; ^3^ Department of Special Treatment, Eastern Hepatobiliary Surgery Hospital, Shanghai, China; ^4^ Institute of Hepatobiliary Surgery, Southwest Hospital, Third Military Medical University, Chongqing, China; ^5^ Department of General Surgery, The First Affiliated Hospital of Zhengzhou University, Zhengzhou, China; ^6^ Department of Interventional Radiology, Chinese PLA General Hospital, Beijing, China; ^7^ Shanghai Cancer Center, Fudan University, Shanghai, China; ^8^ Department of Hepatobiliary Surgery, Nanfang Hospital of Southern Medical University, Guangzhou, China; ^9^ Department of Hepatobiliary Surgery, Xijing Hospital, Xi'an, China; ^10^ Department of Hepato-Pancreato-Biliary Surgery, Beijing Cancer Hospital, Peking University, Beijing, China; ^11^ Department of Radiology, Zhejiang Cancer Hospital, Hangzhou, China; ^12^ Department of Liver Surgery, Renji Hospital, Shanghai Jiaotong University School of Medicine, Shanghai, China; ^13^ Department of Hepatobiliary Surgery, Second Affiliated Hospital of Sun Yat-Sen University, Guangzhou, China; ^14^ Department of Nuclear Medicine, Changhai Hospital, Second Military Medical University, Shanghai, China; ^15^ Department of General Surgery, Ruijin Hospital, Shanghai Jiaotong University School of Medicine, Shanghai, China; ^16^ Department of Medical Oncology, The First Hospital of China Medical University, Shenyang, China; ^17^ Department of Hepatobiliary, Cancer Center, Sun Yat-sen University, Guangzhou, China; ^18^ Department of Liver Transplantation, Shanghai Changzheng Hospital, Shanghai, China; ^19^ Global Medical Affairs, Bayer Healthcare Pharmaceuticals, Montville, NJ, USA; ^20^ Medical Affairs Oncology, Bayer Healthcare Company Ltd., Beijing, China

**Keywords:** sorafenib, unresectable hepatocellular carcinoma, GIDEON, Child-Pugh, Chinese subset

## Abstract

We report data from the final analysis of the Chinese subset of the GIDEON (the Global Investigation of therapeutic DEcisions in hepatocellular carcinoma and Of its treatment with sorafeNib) study, which evaluated the safety and efficacy of sorafenib in Child-Pugh A, B and C patients with unresectable hepatocellular carcinoma (uHCC) in real-life clinical practice. Patient demographics, disease characteristics and treatment history were recorded at enrollment; dose, adverse events (AEs) and efficacy were recorded at follow-up. Of the 338 evaluable patients, 98.5% started on 800 mg/day sorafenib, regardless of their Child-Pugh status. The median treatment duration (21.1 vs. 18.8 weeks) and median overall survival (322 vs 240 days) were longer in patients with Child-Pugh A compared with the Child-Pugh B, progression-free survival were 183 vs. 208 days, respectively). AEs (all grades) were comparable in the Child-Pugh B vs A group (56.3% vs. 50.4%, respectively), moreover, the Child-Pugh B group also had comparable rates of drug-related AEs (35.4% vs. 27.2%, respectively) and serious AEs (25.0% vs. 23.0%, respectively) compared with the Child-Pugh A group. The overall dosing strategy was consistent in Chinese patients across Child-Pugh subgroups. Tolerability and safety data suggest that Child-Pugh B patients might be safely treated with sorafenib. The findings from our study showed that safety profile of sorafenib in terms of rate and type of AEs is similar to the global international GIDEON study as well as other pivotal studies.

## INTRODUCTION

Primary liver cancer (PLC) represents the sixth most common malignancy worldwide and is the third leading cause of cancer-related deaths [1]. Hepatocellular carcinoma (HCC) is the most common type of PLC. Globally, ∼75% of the deaths occurring due to HCC worldwide is expected to rise, due partly to the increasing incidence of hepatitis C [2]. Owing to its large population China has a very high prevalence of PLCs, contributing to more than 50% of all PLCs worldwide, with estimated cases of 293,000 in males and 101,000 in females, as reported in GLOBOCAN 2012 [3, 4]. Data from various studies have reflected that China has the highest prevalence of hepatitis B virus (HBV) carriers in the world and an increasing trend in the rate of hepatitis C virus (HCV) infection. All these factors contribute to a heavy disease burden in China. Although healthcare advancements have been noted in the management of HCC, there has not been any considerable reduction in the mortality over the past few decades. The Ministry of Health of China has also taken several measures to control the disease, but it still remains a health issue of major concern [4–8].

Most HCC patients present unresectable disease (uHCC), wherein surgery is contraindicated. Moreover, survival benefits obtained from medical interventions are controversial. Nonsurgical loco-regional treatment options, such as image-guided ablation and transcatheter arterial chemoembolization (TACE) or use of ^90^Y microspheres (TheraSphere), have limited applicability and may lead to high rates of recurrence [9,10]. Despite the magnitude of the burden of uHCC patients with uHCC still have poor prognosis and limited treatment options. Therefore, further studies are warranted in the field of management of uHCC in terms of treatment morbidity, mortality, and survival.

Sorafenib is an orally bioavailable multitargeted tyrosine kinase inhibitor with potential antiangiogenic and antiproliferative properties that acts by blocking a number of protein kinases[11, 12]. Based on the promising results observed in several clinical trials, sorafenib has been suggested as a first-line therapy in patients with uHCC patients for several years [13–17], and, was approved by the China Food and Drug Administration in 2008 [18]. Two placebo-controlled, Phase III studies (SHARP and Asia-Pacific) acted as milestones in the treatment of patients with uHCC (Child-Pugh A), revealing that sorafenib caused significant improvements in overall survival (OS) [5, 19]. Yao et al, in an analysis concluded that discontinuation of sorafenib treatment for a long period can lead to uHCC disease progression, and, appropriate management of sorafenib-related AEs could result in better clinical outcomes in patients with HCC [20].

GIDEON (the Global Investigation of therapeutic DEcisions in hepatocellular carcinoma and Of its treatment with sorafeNib) is a global, international, prospective, non-interventional study undertaken to fulfill post-approval commitments to licensing agencies for compiling a large, more comprehensive, and robust database of HCC treatment patterns and outcomes among patients with advanced disease [21]. The GIDEON trial evaluated the safety and efficacy of sorafenib in uHCC patients under real-life clinical practice conditions, including patients with Child-Pugh B liver function as well, who were usually excluded from randomized clinical trials [21]. Being one of the largest studies undertaken in patients with uHCC, GIDEON allows the conduct of multiple predefined sub-analyses, focusing on potentially predictive or prognostic factors, including the Child-Pugh score, staging systems, etiology, and prior or concomitant TACE. Results from these sub-analyses would be useful to analyze differences that would influence prognosis and management of HCC, and, thus, will aid physicians in effectively treating HCC to improve patient outcomes [22–25]. Here we report the final analysis of safety and efficacy of sorafenib treatment in the Chinese subset of GIDEON study.

## RESULTS

### Patient disposition and baseline characteristics by Child-Pugh status

During January 2009 and April 2012, a total of 3371 patients from 39 countries of 5 regions were included in the GIDEON study (NCT00812175). The Chinese subset included a total of 345 patients, of which, 338 patients (92.3% were male) were included in the ITT population. The safety (SAF) population included 331 patients. At the start of sorafenib therapy, 74.0% of patients (median age: 50 years) had Child-Pugh A status, 15.1% of patients (median age: 52 years) had Child-Pugh B status, and 0.6% of patients (median age: 58 years) had Child-Pugh C status. Majority of the patients belonged to Eastern Cooperative Oncology Group performance status (ECOG PS) of 0 or 1 (Child-Pugh A: 90%; Child-Pugh B: 66.7%; Child-Pugh C: 50%). Tumor node metastasis (TNM) stage IV was more prominent in Child-Pugh A (41.2%) patients, whereas most of the Child-Pugh B and C patients reported TNM stage III (51% vs. 50%, respectively). Barcelona Clinic Liver Cancer (BCLC) stage C was prominently noted in the majority of the patients (63.2% of patients with Child-Pugh A and 68.6% with Child-Pugh B). Baseline characteristics by Child-Pugh status are summarized in Table 1.

**Table 1:** Baseline characteristics by Child-Pugh status

	Child-Pugh A	Child-Pugh B	Child-Pugh C	Total*
**Number of patients (%)**	250 (74.0)	51 (15.1)	2 (0.6)	338(100)
**Median age (years)**	50.0	52.0	58.0	50.0
**Gender** **Male (%)** **Female (%)**	231 (92.4) 19 (7.6)	47 (92.2) 4 (7.8)	2 (100) 0 (0)	312 (92.3) 26 (7.7)
**ECOG PS (%)** **0 or 1** **≥2**	225 (90) 25 (10)	34 (66.7) 17 (33.3)	1 (50) 1 (50)	286 (84.6) 52 (15.4)
**TNM stage (%)** **I** **II** **III** **IV**	13 (5.2) 28 (11.2) 88 (35.2) 103 (41.2)	0 (0) 2 (3.9) 26 (51.0) 19 (37.3)	0 (0) 0 (0) 1 (50) 1 (50)	13 (3.8) 32 (9.5) 124 (36.7) 137 (40.5)
**BCLC stage (%)** **A** **B** **C** **D**	15 (6.0) 49 (19.6) 158 (63.2) 10 (4.0)	1 (2.0) 8 (15.7) 35 (68.6) 4 (7.8)	0 (0) 0 (0) 0 (0) 2 (100)	18 (5.3) 59 (17.5) 210 (62.1) 18 (5.3)

BCLC, Barcelona Clinic Liver Cancer; ECOG PS, Eastern Cooperative Oncology Group performance status; TNM, tumor node metastasis.

* Includes 35 non-evaluable patients.

### Sorafenib dosing and duration

Overall, the majority of patients received the recommended initial dose of 800 mg (98.5%); the median daily dose was 800 mg. These were consistent across all Child-Pugh subgroups. The duration of treatment was slightly longer in Child-Pugh A (21.1 weeks) patients than in Child-Pugh B patients (18.8 weeks) (Table 2).

**Table 2:** Sorafenib administration by Child-Pugh status

	Child-Pugh A (*N*=250)	Child-Pugh B (*N*=51)	Child-Pugh C (*N*=2)	Total* (*N*=338)
**Initial dose of 800 mg (%)**	245 (98.0)	51 (100.0)	2 (100.0)	333 (98.5)
**Initial dose of 400 mg (%)**	5 (2.0)	0	0	5 (1.5)
**Median daily dose (mg)**	800	800	800	800
**Median treatment duration (weeks)**	21.1	18.8	63.3	21.1

* Includes 35 non-evaluable patients.

**Figure 1:**
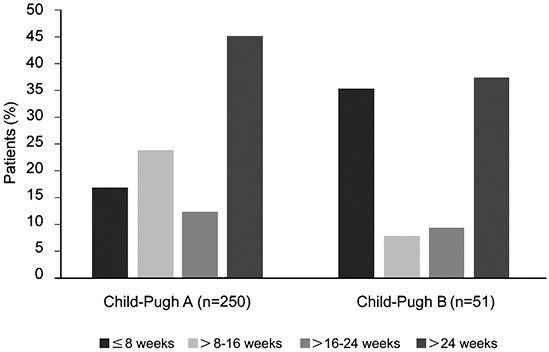
Duration of treatment by Child-Pugh status

The majority of patients in the Child-Pugh B group received sorafenib for <8 weeks. However, a considerable percentage of patients also received sorafenib for >24 weeks, though the number was fewer than that in the Child-Pugh A group. The percentages of patients treated for >28 weeks in the Child-Pugh A and B groups were 40.8% and 31.4%, respectively (Figure 1).

### Safety assessment

**Figure 2:**
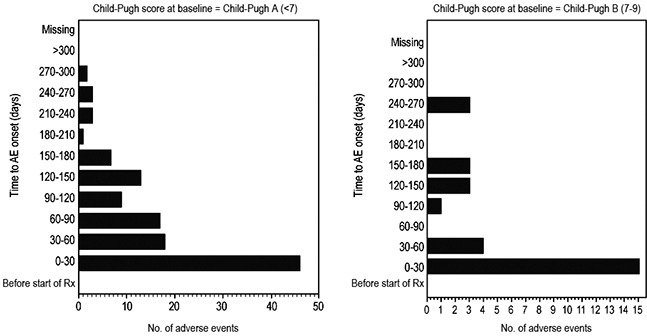
Time to onset of adverse events greater than grade 1 by Child-Pugh status

Based on the SAF dataset, a comparison of the incidence of AEs and serious AEs in the Child-Pugh A and B subgroups is presented in Table 3A. No AEs were observed in the Child-Pugh C group. AEs (all grades) were comparable in the Child-Pugh B group compared with the Child-Pugh A group (56.3% vs. 50.4%, respectively). Similarly, Child-Pugh B group also had comparable rates of drug-related AEs (35.4% vs. 27.2%, respectively) and serious AEs (25.0% vs. 23.0%, respectively) as compared with the Child-Pugh A group. The most common drug-related AEs overall were diarrhea, hand-foot skin reaction (HFSR), and rash/desquamation. No unexpected AEs were observed in patients with liver dysfunction (Table 3B). The incidence of drug-related HFSR was higher in the Child-Pugh A group than in the Child-Pugh B group (20.7% vs 14.6%, respectively), although incidence of diarrhea was higher in the Child-Pugh B group than in the Child-Pugh A group (14.6% vs 10.2%). The rates of drug-related AEs, calculated as the number of events per patient-year, in the Child-Pugh A and B groups are presented in Table 3C. The time to onset of AEs greater than grade 1 in the Child-Pugh A group was comparable to that in the Child-Pugh B group, with the majority of AEs occurring within the first 30 days of treatment in both groups (Figure 2). Overall, comparable rates of treatment-emergent AEs were reported in Child-Pugh B and Child-Pugh A patients. Discontinuation of treatment due to AEs was slightly more common in Child-Pugh B than in Child-Pugh A patients (10.4% vs 8.9%). Also, the number of treatment-emergent deaths occurring up to 30 days after the last sorafenib dose was comparable in Child-Pugh B patients and Child-Pugh A patients.

**Table 3A:** Treatment-emergent adverse events (AEs) by Child-Pugh status

%	Child-Pugh A (*N*=246)	Child-Pugh B (*N*=48)	Child-Pugh C (*N*=2)	Total* (*N*=331)
**AEs (all grades)**	124 (50.4)	27 (56.3)	0	167 (50.5)
**Drug-related AEs (all grades)**	67 (27.2)	17 (35.4)	0	95 (28.7)
Serious AEs (all grades)**	58 (23.6)	12 (25.0)	0	77 (23.3)
**Drug-related serious AEs (all grades)**	0	1 (2.1)	0	1 (0.3%)
**All grade 3 or 4**	14 (5.7)	4 (8.3)	0	20 (6.0)
**Drug-related grade 3 or 4**	9 (3.7)	2 (4.2)	0	12 (3.6)
**AEs resulting in permanent discontinuation of sorafenib**	22 (8.9)	5 (10.4)	0	29 (8.8)
Deaths***	127 (51.6)	27 (56.3)	0	166 (50.2)

* Includes 35 non-evaluable patients.

** Any AE occurring at any dose that results in any of the following consequences: death; life-threatening conditions; hospitalization or prolongation of existing hospitalization; persistent or significant disability/incapacity; congenital anomaly/birth defect; and medically important event.

*** Treatment-emergent deaths occurring up to 30 days after the last sorafenib dose.

**Table 3B:** Drug-related adverse events by Child-Pugh status

%	Child-Pugh A (*N*=246)	Child-Pugh B (*N*=48)	Child-Pugh C (*N*=2)
**Diarrhea**	25 (10.2)	7 (14.6)	0
**HFSR**	51 (20.7)	7 (14.6)	0
**Fatigue**	1 (0.4)	0	0
**Rash/desquamation**	8 (3.3)	3 (6.3)	0
**Hypertension**	7 (2.8)	1 (2.1)	0
**Alopecia**	6 (2.4)	2 (4.2)	0
**Nausea**	0	1 (2.1)	0
**Pain, abdomen NOS**	2 (0.8)	0	0

HFSR, hand-foot skin reaction; NOS, not otherwise specified

**Table 3C:** Rate of drug-related adverse events (AEs) by Child-Pugh status

Rate (events per patient-year*)	Child-Pugh A (*N*=246)	Child-Pugh B (*N*=48)	Child-Pugh C (*N*=2)	Total** (*N*=331)
**Any AE**	0.43	0.64	0	0.46
**Diarrhea**	0.16	0.26	0	0.17
**HFSR**	0.33	0.26	0	0.30
**Liver dysfunction**	0	0	0	0

* Rate calculation based on treatment-emergent AEs with .10% incidence and 365.25 days per year.

** Includes 35 non-evaluable patients.

HFSR, hand-foot skin reaction.

### Efficacy outcomes

**Figure 3:**
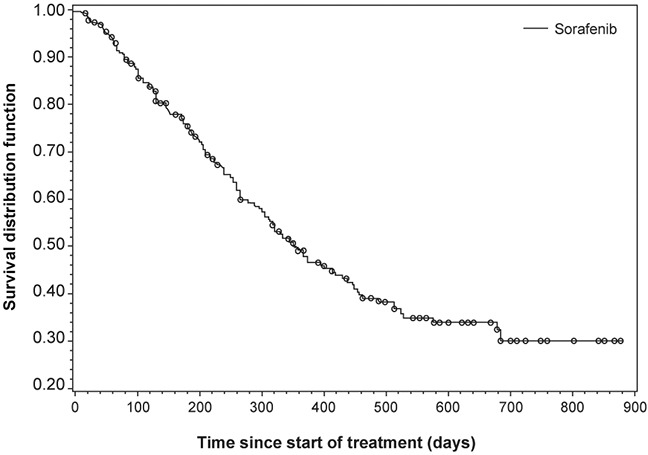
Overall survival in the Chinese subset

**Figure 4:**
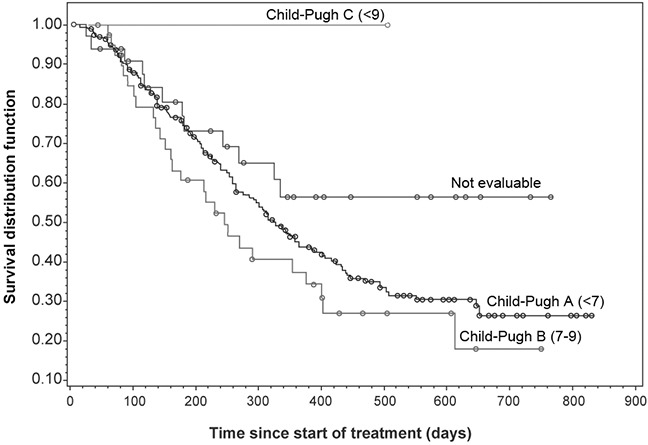
Overall survival by Child-Pugh score

Median OS was 322 days in the Chinese subset (Figure 3). The OS was longer in Child-Pugh A patients than in Child-Pugh B patients (Table 4, Figure 4). Child-Pugh B patients showed a similar progression-free survival (PFS) to that of Child-Pugh A patients. Median time to progression (TTP) could not be compared between subgroups due to the high censoring rate in the Child-Pugh B and C groups.

**Table 4:** Survival outcomes by Child-Pugh status

	Child-Pugh A	Child-Pugh B	Child-Pugh C
**Overall survival** **Number failed** **Number censored** **Median (days)**	127 (50.8%) 123 (49.2%) 322	27 (52.9%) 24 (47.1%) 240	– 2 (100%) –
**Progression-free survival** **Number failed** **Number censored** **Median (days)**	173 (69.2%) 77 (30.8%) 183	31 (60.8%) 20 (39.2%) 208	– 2 (100%) –
**Time to progression** **Number failed** **Number censored** **Median (days)**	85 (34.0%) 165 (66.0%) 214	4 (7.8%) 47 (92.2%) –	– 2 (100%) –

## DISCUSSION

Previously conducted large clinical trials enrolled Child-Pugh class A patients, and thus, the viability of sorafenib treatment in patients with uHCC and poor liver function was largely unknown[5,19]. GIDEON is of considerable clinical interest and relevance, as the study provides an opportunity to assess a wider patient population as compared with randomized clinical trials. Observations from this study will aid physicians in effectively treating HCC and improving patient outcomes. To our knowledge, GIDEON is amongst the largest study undertaken in patients with HCC involving 3371 patients from 39 countries of 5 regions [21].

The results of the Chinese subset of GIDEON reveal that patient demographics in China are largely in line with the previous global GIDEON report in terms of gender, staging, Child-Pugh status, and performance status [25]. However, in our study, patients appeared to be younger, and most of them belonged to BCLC Stage C (62.1%), in whom sorafenib could be considered an appropriate treatment option [26].

Several studies have indicated that sorafenib is beneficial in Child-Pugh B patients, with an acceptable safety profile [25, 27–32]. In our study, Child-Pugh status did not appear to influence the approach to sorafenib dosing. A considerable percentage of Chinese patients in the Child-Pugh B group received sorafenib for >28 weeks. In contrast to the global report, almost all Chinese patients received the recommended initial daily dose of sorafenib (800 mg/day), suggesting that sorafenib was well tolerated in Chinese patients. Notably, the median treatment duration was 63.3 weeks in 2 Chinese Child-Pugh C patients, which might imply that patients with liver dysfunction could still receive sorafenib therapy. However, the extremely small sample size in this subgroup does not allow us to draw a definite conclusion and hence more studies are warranted.

With the exception that fatigue was not a common AE in Chinese patients, the safety profile was largely consistent with that in the global report, with diarrhea, HFSR, and rash/desquamation being the most commonly reported drug-related AEs. Although the Child-Pugh B group had most of the drug-related AEs, the Child-Pugh A group had more HFSR cases. Comparable rates of treatment-emergent AEs were reported among Child-Pugh B and Child-Pugh A patients. Discontinuation of treatment due to AEs was slightly more common in Child-Pugh B than in Child-Pugh A patients (10.4% vs 8.9%). The majority of AEs greater than grade 1 occurred within the first 30 days of treatment in both Child-Pugh A and B groups. Overall, most commonly reported drug-related AEs across Child-Pugh subgroups were comparable in both type and incidence.

Consistent with previous reports, OS was longer in Child-Pugh A patients compared with Child-Pugh B or C patients; suggesting that Child-Pugh status is a strong prognostic indicator of OS in uHCC patients, regardless of treatment with sorafenib. PFS was similar between Child-Pugh A and B patients. As shown in Table 4, many patients in the Child-Pugh B (TTP analysis) and C (OS, PFS, and TTP analyses) groups may have been lost to follow-up. These findings are in line with the previously conducted SHARP and Asia-Pacific study. The SHARP trial enrolled 602 patients and showed prolonged median OS (10.7 vs. 7.9 months; *P*<.001) and prolonged time to radiological disease progression (5.5 vs. 2.8 months; *P*<.001) for sorafenib compared with placebo [19]. Similarly, the Asia-Pacific study demonstrated that sorafenib had a substantially longer median OS (6.4 vs 4.2 months) and median TTP (2.8 vs 1.4 months) over placebo [5].

Our study has certain limitations. First, being an observational study, it is subjected to known and unknown confounders and biases. Second, lack of randomization and a placebo or a comparative group may change the study outcomes. Third, statistical limitation of our data must be considered before drawing any definitive conclusion. Owing to all the above factors, it becomes mandatory that the implications of this observational study must be interpreted with caution. Further prospective trials are much needed to evaluate the efficacy and safety of sorafenib in Child-Pugh class subgroups, particularly in those patients with less compromised liver function. Nevertheless, the primary strength of our analysis to observe both practice patterns and the safety of an intervention in real-life practice should not be ignored.

The GIDEON study has now been completed, fulfilling a licensing commitment to assess the safety of sorafenib in clinical practice. Our study results suggest that sorafenib at 800 mg/day appears to be safe and better tolerated in Chinese clinical setting. Child-Pugh status was found to be a strong prognostic indicator of OS in uHCC patients. Tolerability and safety data suggest that Child-Pugh B patients might be safely treated with sorafenib. The findings from our study showed that safety profile of sorafenib in terms of rate and type of AEs is similar to GIDEON study, in Chinese uHCC patients with Child-Pugh B, as well other pivotal GIDEON subset studies [21, 33, 34].

## MATERIALS AND METHODS

### Study design

GIDEON was the largest, international, prospective, open-label, non-interventional study that presented data from a global perspective. Design and rationale of the GIDEON study have already been published by Lencioni et al [21]. As a non-interventional study, assignment to a particular therapeutic strategy was not mandated by the study protocol but imitated the current practice of participating physicians. The decision to include a patient in the study was separate from the treatment decision. The final analysis was conducted upon completion of the 12-month follow-up assessment [21].

### Study objectives

The primary objective was to evaluate the safety of sorafenib under real-life clinical setting; secondary objectives included evaluation of efficacy (OS, PFS, TTP, response rate, and rate of stable disease) and duration of therapy; regional and global methods of patient evaluation, diagnosis, and follow-up; evaluation of comorbidities and their influence on treatment and outcome; and evaluation of practice patterns of the physicians involved in the care of these patients. Computed tomography (CT) and other radiographic methods were used by the physicians for assessing the tumor at baseline, at follow-up visits, or at a frequency determined by the treating physician.

### Patient population

The inclusion criteria were similar to that of the principal GIDEON trial [21]. Patients were eligible for participating in GIDEON if they were ≥18 years of age; were diagnosed histologically, cytologically, or radiographically with uHCC; had a life expectancy of >8 weeks; had not undertaken previous sorafenib treatment; were candidates for systemic therapy; and in whom the decision to be treated with sorafenib had been made by their physician (radiographic diagnosis required the typical observations of HCC by a radiographic method, i.e., multidimensional dynamic CT, CT hepatic arteriography/CT arterial portography, or MRI). Moreover, the patients' physicians must be willing to duly complete and submit all case report forms and must be ready to submit to a site audit with verification of the source documents along with all the other related records/testimonials and validation of the reported data. Exclusion criteria were based on the local product information for sorafenib.

### Informed consent

Signed patient consent form and eligibility criteria form were completed for all patients who were included in the study and were approved by local Ethics Review Board. All patients' data were kept anonymous. All the participants received a written explanation of the study and provided written informed consent before participating.

### Ethical considerations

This study followed the ethical principles approved by the local institutional review board, ethics committees, and health authorities according to Good Clinical Practice and local laws and regulations in China, as well as established recommendations and regulations pertaining to non-interventional and post-authorization safety studies [26]. All procedures followed were in accordance with the ethical standards of the responsible committee on human experimentation (institutional and national) and with the Helsinki Declaration of 1975, as revised in 2008.

### Data collection

Baseline characteristics were recorded, and information regarding AEs, dosing, and outcomes was collected during follow-up visits. AEs and other safety parameters, including blood pressure, Child-Pugh grade, and ECOG PS, were summarized using the safety population. All data were collected using case report forms at study entry and at the initiation of sorafenib treatment, and then at intervals normally decided by the prescribing physician (usually ≥6 weeks or ≥12 weeks), or until patient's death, withdrawal, or loss to follow-up, or if significant changes in a patient's disease were observed [17]. Analyses of the final data were conducted to evaluate dosing patterns, AEs, and outcomes [21].

### Statistical analyses

Patients who received at least one dose of sorafenib and underwent at least one follow-up assessment were evaluable for safety and efficacy. All AEs were graded according to the National Cancer Institute Common Terminology Criteria for Adverse Events version 3.0. Descriptive statistics, such as frequency, proportion, mean, median, standard deviation, 95% confidence intervals, and quartiles, were calculated for all baseline, safety, and efficacy data. Kaplan–Meier estimates were calculated for the OS, PFS, and TTP efficacy end points.

The recruitment number was based on an overall sample of 3000 patients, a number considered sufficient for comprehensive evaluation of safety for the overall population, as well as planned subgroup analyses [13]. No *P*-values are presented as all data are summarized using descriptive statistics.
